# *Listeria monocytogenes* occurrence and genetic diversity within the pre-packaging area of an Italian cold-smoked salmon processing plant

**DOI:** 10.3389/fmicb.2026.1917424

**Published:** 2026-09-21

**Authors:** Michela Berta, Pierluigi Di Ciccio, Maria Teresa Bottero, Daniele Pattono, Giacomo Manacorda, Alessandra Dalmasso

**Affiliations:** Department of Veterinary Sciences, University of Turin, Grugliasco, TO, Italy

**Keywords:** cold-smoked salmon, food safety, genetic diversity, *Listeria monocytogenes*, ready-to-eat

## Abstract

*Listeria monocytogenes* is a major concern in ready-to-eat (RTE) food production, particularly in cold-smoked salmon, which has been linked to several listeriosis outbreaks. This study investigated the occurrence and genetic diversity of *L. monocytogenes* within the pre-packaging area of an Italian cold-smoked salmon processing plant. From March 2023 to December 2023, 69 samples were collected, including 44 food contact surfaces (FCS), 15 non-food contact surfaces (NFCS), and 10 food samples (F). Microbiological analyses were conducted according to the standard qualitative method EN ISO 11290:2017, and suspected colonies were confirmed by MALDI-TOF mass spectrometry. Isolates were further characterized by multilocus sequence typing (MLST). As results, 18 samples showed the presence of *L. monocytogenes*, corresponding to an overall detection rate of 26.09%. The highest positivity rate was observed among NFCS (86.67%), followed by F (10%) and FCS (9.09%). MLST grouped isolates into five STs: ST5 (38.89%), ST9 (27.78%), ST6 (16.67%), ST7 (11.11%), ST14 (5.56%). ST5 and ST9 were the predominant STs and were repeatedly detected throughout the sampling period. These findings highlighted the importance of environmental monitoring, strengthened sanitation measures, and molecular typing to improve *L. monocytogenes* control in cold-smoked salmon processing plants.

## 1 Introduction

*Listeria monocytogenes* is a gram-positive bacterium responsible for listeriosis, a serious foodborne disease potentially fatal for high-risk populations including immuno-compromised patients, elderly, and infants. Symptoms of listeriosis can range from mild, flu-like signs such as fever, muscle aches, and gastrointestinal disturbances to more severe and life-threatening complications like meningitis and endocarditis. Furthermore, in pregnant women, listeriosis increases the risk of miscarriage and pre-term birth (Koopmans et al., 2023; Møretrø et al., 2025). In 2024, the European listeriosis fatality rate reached 15.6%, with a notification rate of 0.69 cases per 100,000 inhabitants and a total of 3,041 confirmed cases, 238 of which were reported in Italy. Despite the relatively low incidence (0.40 per 100,000 people), Italy was involved in at least six listeriosis outbreaks between 2019 and 2024 (The European Union one health 2024 Zoonoses report, 2025). In food production, the management of *L. monocytogenes* is a significant concern and requires considerable efforts (Ferreira et al., 2014). The accidental introduction of this pathogen into food facilities may occur through various routes, including raw materials, personnel, contaminated utensils and pests. Once established in the processing environment, *L. monocytogenes* is able to withstand the various stresses typical of food production, such as refrigeration, high salt concentrations, acidic pH and low-oxygen condition (Bucur et al., 2018; Osek et al., 2022) In addition, the capacity to adhere to surfaces and form biofilm significantly enhances resistance to routine cleaning and disinfection procedures (Finn et al., 2023; Myintzaw et al., 2023). The presence of *L. monocytogenes* within food production environments may last extended periods, ranging from months to years (Carpentier and Cerf, 2011). Inevitably, this long-term persistence led to substantial economic losses for food producers and an increased likelihood for cross-contamination (Belias et al., 2024; Stessl et al., 2022). Listeriosis transmission has been mainly associated with the ingestion of contaminated ready-to-eat (RTE) foods (Kurpas et al., 2018). Among these, vacuum-packaged cold-smoked salmon is a widely consumed RTE that has been recently implicated in multiple listeriosis outbreaks (European Centre for Disease Prevention and Control European Food Safety Authority, 2018; European Centre for Disease Prevention and Control, European Food Safety Authority, 2023; European Centre for Disease Prevention and Control European Food Safety Authority, 2024).

This RTE product is characterized by high water activity (aw > 0.96) and a pH ranging from 6 to 6.5, conditions that favor the growth of *L. monocytogenes* (Løvdal, 2015). However, the susceptibility to pathogen contamination is closely related to its production process. Firstly, the technological procedures employed, such as salting, cold smoking, vacuum packaging, and low storage temperatures, could be insufficient to ensure the absence of *L. monocytogenes* in the finished product (Geraldo et al., 2024; Leong et al., 2015). In addition, throughout cold-smoked salmon production, the food matrix is subjected to several handling steps (bone removal, skinning, and weighing) and comes into contact with numerous processing surfaces (blades, conveyor belts, tables, and trolleys) that could serve as sources of *L. monocytogenes* (Gil et al., 2024; Jami et al., 2014).

Consequently, strict control of the processing environment is crucial to mitigate the risk of contamination. Accurate environmental monitoring allows food producers to detect the pathogen early, identifying contamination hotspots and assessing the effectiveness of sanitation procedures (Gil et al., 2024; Gupta and Adhikari, 2022; Jordan et al., 2018). Furthermore, the proper application of Good Manufacturing Practices (GMPs) is essential to contain the spread of *L. monocytogenes* across different production areas (Geraldo et al., 2024).

In this context, culture-based methods are extensively and effectively used to determine the presence of *L. monocytogenes* in food industry monitoring programs (Jordan et al., 2018).

However, these approaches are limited to the detection and enumeration of the pathogen and do not provide information on the genetic diversity of the strains circulating within the production environment. Therefore, to further strengthen surveillance strategies, traditional microbiological analyses are often combined with molecular methodologies for strains characterization (Carpentier and Cerf, 2011; Gupta and Adhikari, 2022; Jordan et al., 2018). Among these, Multilocus sequence typing (MLST) is a widely adopted method that assigns *L. monocytogenes* isolates to specific sequence types (STs) based on allelic profiles of seven housekeeping genes (Ragon et al., 2008).

Several studies have shown that certain STs are overrepresented in food-processing environments, suggesting an enhanced capacity of these types to adapt to industrial stress conditions. Among these, ST9 and ST121 are frequently isolated from surfaces of food processing facilities (Alvarez-Molina et al., 2021; Maury et al., 2016). In contrast, ST1, ST2, ST4, and ST6 are predominantly associated with clinical samples and have been previously described as hypervirulent (Maury et al., 2016).

So far, numerous studies have assessed the distribution and genetic diversity of *L. monocytogenes* in meat and dairy processing plants. Conversely, investigations into RTE seafood production environments remain scarce. However, the recent listeriosis outbreaks associated with the consumption of contaminated cold-smoked salmon highlights the need for increased attention to the production of this RTE food. For these reasons, the aim of this study was to investigate the occurrence of *L. monocytogenes* in the pre-packaging area of an Italian cold smoked salmon processing plant and assess the diversity of isolated strains by MLST.

## 2 Materials and methods

### 2.1 Cold smoked salmon processing plant description

The Italian salmon processing plant involved in this study is a long-established enterprise with over two decades of experience, operating on an industrial scale.

The raw material, supplied weekly, is sourced mainly from Norwegian and Scottish fish slaughterhouses. Atlantic salmon (*Salmo salar*), received whole, pre-gutted, and under refrigerated conditions is firstly deheaded; subsequently fins and tail are removed by operators, and then conveyed to the filleting machine. Afterwards, salmon filets undergo a long salting process lasting approximately 20 h. This facility employs the dry curing method, carried out manually and exclusively with sea salt. After washing, product is subjected to cold smoking treatment at 30 °C for 3 h, followed by 20-h resting period in the drying chamber.

Smoked salmon filets are ready to be transferred to the pre-packaging area, where they will undergo trimming, skin removal and slicing. Finally, the sliced product is transferred to the packaging area, where it is weighed and finally vacuum packaged.

Each processing area operates daily from 6 a.m. to 10 p.m., while cleaning and sanitation procedures are scheduled overnight immediately after production ends.

### 2.2 Samples collection

According to the plant’s self-monitoring plan, the product pre-packaging area consistently showed the highest rate of *L. monocytogenes* positivity and was therefore selected as the target area for sampling. Sampling sites corresponded to those routinely monitored within the existing self-monitoring-plan.

They were selected considering the processing workflow and the areas identified as having a higher potential risk for *L. monocytogenes* contamination. Sampling points are depicted in Figure 1.

**Figure 1:**
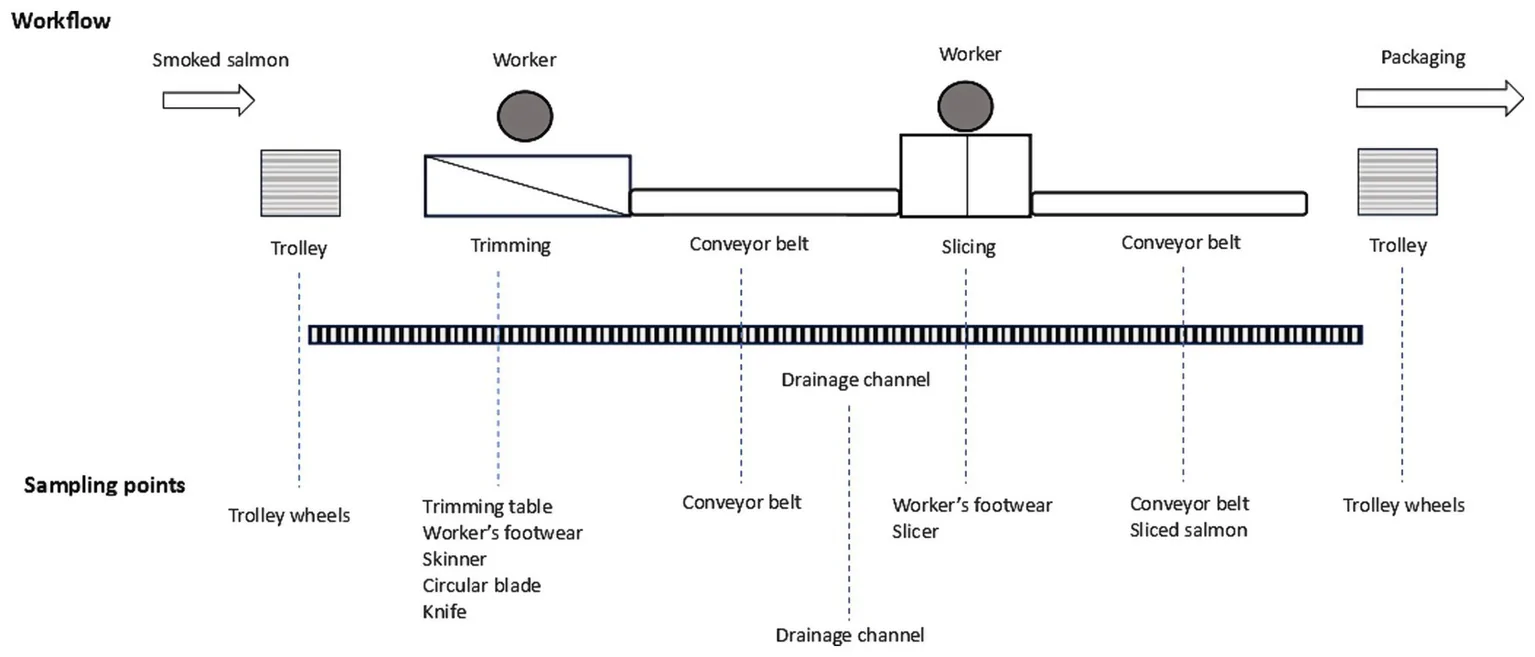
Workflow of the cold-smoked salmon pre-packaging area and sampling points. Smoked salmon fillets arrive on trolleys and are transferred to trimming table, where the skin is removed by a skinner machine and operators eliminate the brown muscle tissue using a knife and a circular blade. The processed fillets are then conveyed to an automatic slicing machine. Finally, the sliced cold-smoked salmon is transferred to the packaging area.

Processing plant was visited three times, in March 2023, July 2023, and December 2023 to cover the periods of medium, low, and high production intensity, respectively. Each sampling session was conducted at 10:00 a.m. during normal working conditions. Sample collection included food contact surfaces (FCS), non-food contact surfaces (NFCS), and food matrix (F).

Selected FC and NFC surfaces were sampled using sterile sponges by thoroughly swabbing the accessible surface area of each sampling site. F samples were obtained by collecting 25 g of salmon directly from the conveyor belt, immediately after the slicing step. Samples were transported to the laboratory under refrigerated conditions and were analyzed within 24 h.

In total, 69 samples were collected, 23 for each sampling visit. They included 44 FCS, 15 NFCS and 10 F samples (Tables 1, 2).

**Table 1:** Overview of collected and *L. monocytogenes*-positive samples by category and sampling period.

Sampling period	Collected samples				Positive samples			
	NFCS	FCS	F	Total	NFCS	FCS	F	Total
March	5	14	4	23	4	3	0	7
July	5	16	2	23	4	0	0	4
December	5	14	4	23	5	1	1	7
Total	15	44	10	69	13	4	1	18

**Table 2:** Overview of total analysed samples.

Sample	Classification	Date	*L. monocytogenes*	Sequence type
Slicer	FCS	March 2023		
Circular blade	FCS	March 2023		
Conveyor belt	FCS	March 2023	+	ST5
Conveyor belt	FCS	March 2023	+	ST5
Trimming table	FCS	March 2023	+	ST6
Skinner	FCS	March 2023		
Sliced salmon	F	March 2023		
Sliced salmon	F	March 2023		
Worker’s footwear	NFCS	March 2023	+	ST5
Trolley wheels	NFCS	March 2023	+	ST6
Skinner	FCS	March 2023		
Trimming table	FCS	March 2023		
Circular blade	FCS	March 2023		
Knife	FCS	March 2023		
Conveyor belt	FCS	March 2023		
Conveyor belt	FCS	March 2023		
Slicer	FCS	March 2023		
Trimming table	FCS	March 2023		
Trolley wheels	NFCS	March 2023		
Worker’s footwear	NFCS	March 2023	+	ST9
Sliced salmon	F	March 2023		
Sliced salmon	F	March 2023		
Drainage channel	NFCS	March 2023	+	ST14
Slicer	FCS	July 2023		
Circular blade	FCS	July 2023		
Trimming table	FCS	July 2023		
Conveyor belt	FCS	July 2023		
Conveyor belt	FCS	July 2023		
Skinner	FCS	July 2023		
Knife	FCS	July 2023		
Trimming table	FCS	July 2023		
Sliced salmon	F	July 2023		
Worker’s footwear	NFCS	July 2023	+	ST5
Trolley wheels	NFCS	July 2023		
Skinner	FCS	July 2023		
Trimming table	FCS	July 2023		
Circular blade	FCS	July 2023		
Conveyor belt	FCS	July 2023		
Conveyor belt	FCS	July 2023		
Slicer	FCS	July 2023		
Sliced salmon	F	July 2023		
Knife	FCS	July 2023		
Conveyor belt	FCS	July 2023		
Worker’s footwear	NFCS	July 2023	+	ST9
Trolley wheels	NFCS	July 2023	+	ST7
Drainage channel	NFCS	July 2023	+	ST7
Skinner	FCS	December 2023		
Trimming table	FCS	December 2023	+	ST5
Circular blade	FCS	December 2023		
Knife	FCS	December 2023		
Conveyor belt	FCS	December 2023		
Conveyor belt	FCS	December 2023		
Slicer	FCS	December 2023		
Sliced salmon	F	December 2023	+	ST5
Sliced salmon	F	December 2023		
Worker’s footwear	NFCS	December 2023	+	ST5
Trolley wheels	NFCS	December 2023	+	ST9
Drainage channel	NFCS	December 2023	+	ST6
Slicer	FCS	December 2023		
Circular blade	FCS	December 2023		
Conveyor belt	FCS	December 2023		
Conveyor belt	FCS	December 2023		
Trimming table	FCS	December 2023		
Skinner	FCS	December 2023		
Knife	FCS	December 2023		
Sliced salmon	F	December 2023		
Sliced salmon	F	December 2023		
Worker’s footwear	NFCS	December 2023	+	ST9
Trolley wheels	NFCS	December 2023	+	ST9

### 2.3 *Listeria monocytogenes* isolation, confirmation and characterization

Microbiological analyses were conducted following the qualitative standard method EN ISO 11290-1:2017. The methodology included two enrichment steps in half-Fraser and Fraser broth to enhance the recovery of *L. monocytogenes*, including samples with low bacterial loads. Enriched samples were plated onto Oxoid™ Brilliance™ Listeria Agar (ISO) (CM1212B; Thermo Scientific Oxoid, Basingstoke, UK) and incubated for 48 h at 37 °C. Presumptive *L. monocytogenes* colonies, identified by their blue-green coloration and characteristic halo, were subjected to species confirmation by Matrix-Assisted Laser Desorption Ionization Time-of-Flight Mass Spectrometry (MALDI-TOF MS) using the Bruker Biotyper system (Bruker Daltonics, Bremen, Germany).

Genomic DNA of confirmed *L. monocytogenes* strains was extracted using DNeasy Blood & Tissue Kit (Qiagen, Hilden, Germany), according to manufacturer’s instructions.

MLST was performed according to the method previously described by Ragon et al. (2008). The seven housekeeping genes included in the MLST scheme (*abcZ, bglA, cat, dapE, dat, ldh,* and *lhkA*) were amplified by conventional PCR using the corresponding primers.

PCR products were verified by electrophoresis on a 1.5% agarose gel and purified using ExoSAP-IT™ (Applied Biosystems, Foster City, CA, USA). Cycle sequencing reactions were performed separately for forward and reverse strands using the BigDye® Terminator v3.1 Cycle Sequencing Kit (Applied Biosystems, Foster City, CA, USA), according to the manufacturer’s instructions, and the same primers used for PCR amplification. In each reaction, *L. monocytogenes* ATCC 19115 and sterile nuclease-free water were included as positive and negative control, respectively.

Following cycle sequencing, the extension products were purified to remove unincorporated dye terminators and analyzed by Sanger sequencing with the Applied Biosystems SeqStudio™ Genetic Analyzer (Applied Biosystems, Foster City, CA, USA). In each reaction, *L. monocytogenes* ATCC 19115 and sterile nuclease-free water were included as positive and negative control, respectively.

The obtained chromatograms were quality-checked and edited using FinchTV version 1.4. Forward and reverse sequences were subsequently assembled to generate consensus sequences using MEGA version 12. The resulting sequences were compared with the MLST database available through the Institut Pasteur platform (https://bigsdb.pasteur.fr/listeria/) to assign allele numbers, and the corresponding ST was determined based on the allelic profile.

Strains were deposited at the Turin University Culture Collection (TUCC) integrated in the Italian national node (MIRRI-IT) of the Research Infrastructure MIRRI-ERIC.

### 2.4 Statistical analysis

Statistical analyses were performed using R software version 4.5.1. Differences in the proportion of *L. monocytogenes*-positive samples among sampling periods (March, July, December) and sample categories (FCS, NFCS, F) were assessed using Fisher’s exact test. A two-sided *p*-value < 0.05 was considered statistically significant.

## 3 Results and discussion

This study aimed to investigate the prevalence of *L. monocytogenes* in the pre-packaging area of an Italian cold-smoked salmon processing plant and to assess the genetic diversity of isolates.

A total of 69 samples were collected over three samplings visits, of which 18 (26.09%) were positive for the presence of *L. monocytogenes* (Table 1). The highest positivity rates were observed during the sampling visits conducted in March 2023 and December 2023. In both periods, the prevalence of *L. monocytogenes* was 30.43% (7/23), while the sampling performed in July 2023 showed a lower prevalence of 17.39% (4/23) (Table 1). Although higher positivity rates were observed during March and December compared with July, differences among sampling visits were not statistically significant (Fisher’s exact test, *p* = 0.49). Nevertheless, the variation in the number of positive samples might be associated with differences in production intensity. Indeed, in the visited smoked salmon processing plant, March and December correspond to periods of increased production demand, characterized by higher personnel traffic, more intensive equipment use, and larger volumes of raw materials handled. These conditions could increase the likelihood of environmental contamination and may have contributed to the observed variability in *L. monocytogenes* prevalence, thereby representing additional challenges to effective pathogen control within the processing environment.

Moreover, the prevalence of *L. monocytogenes* observed in this study is consistent with previous investigations conducted in seafood processing facilities. Wulff et al. (2006), monitoring four Danish smokehouses over a six-month period (78–105 samples per facility), reported *L. monocytogenes* prevalences ranging from 16 to 32%. Leong et al. (2015), who surveyed eight Irish seafood processing plants over a 20-month period (22–84 samples per facility), found considerably lower positivity rates (<4.3%) in most facilities, although one plant exhibited a prevalence of 33.3%. It should be noted, however, that the studies mentioned above assessed *L. monocytogenes* prevalence across different stages of the production process, including raw fish handling, processing and packaging areas. Conversely, the prevalence reported in the present study was specifically observed at the pre-packaging stage, which represents a critical point in the production chain. At this stage, the product will not undergo any further treatment capable of reducing *L. monocytogenes* contamination, while subsequent manual handling and packaging operations may contribute to an increased risk of cross-contamination. Therefore, these results highlight the need for stringent hygiene practices and effective environmental monitoring and sanitation procedures, particularly during the final stages of processing, to minimize the risk of contamination in finished products.

Of the 18 samples tested positive, the majority of them originated from NFCS (13/15; 86.67%), followed by F (1/10; 10.0%) and (4/44; 9.09%) of FCS (Tables 1, 2). Statistical analysis revealed a significantly greater likelihood of *L. monocytogenes* detection among NFCS compared with FCS (*p* < 0.001) and F (*p* < 0.001).

Regarding NFCS, *L. monocytogenes* was repeatedly detected in workers’ footwear, drainage channel and trolley wheels across all three sampling visits (Tables 2, 3).

**Table 3:** STs distribution during samplings.

Source	March	July	December
Workers’ footwear (NFCS)	ST5	ST5	ST5
	ST9	ST9	ST9
Trolley wheels (NFCS)	ST6	ST7	ST9
	–	–	ST9
Drainage channel (NFCS)	ST14	ST7	ST6
Conveyor belt (FCS)	ST5	–	–
	ST5		
Trimming table (FCS)	ST6	–	ST5

Sliced salmon (F)	–	–	ST5

Footwear and their soles may act as microbial niches due to constant contact with wet floors and the potential accumulation of organic residues. For these reasons, Food Business Operators (FBOs) should ensure frequent and effective cleaning and sanitation of workers’ footwear, particularly when staff move between different production areas. The replacement of worn or damaged footwear should be prioritized, as deteriorated soles (e.g., with cracks or crevices) may facilitate *L. monocytogenes* adherence (Belias et al., 2022). Notably, in the visited smoked salmon processing plant, footwear sanitation stations were located merely at the entrance to the processing environment. This configuration may have limited the frequency of footwear sanitization during working hours, particularly when moving between different production areas. Therefore, the installation of additional sanitation points, together with the implementation of targeted training programs to increase awareness of personal hygiene practices, should be considered (Tuytschaever et al., 2023).

Concerning the pathogen occurrence in drainage system, it is noted that drain offers an ideal habitat for microbial proliferation due to the accumulation of wastewater and organic debris. If not properly controlled, it may facilitate the dissemination of pathogen through aerosols, water flow, staff movement and equipment transfer residues (Ferreira et al., 2014; Gil et al., 2024).

Trolleys are mobile units widely used in cold-smoked salmon production to transport fish filets between processing areas, and they have frequently been identified as vectors for the spread of *L. monocytogenes* and contributing to its long-term persistence within the processing environment (Belias et al., 2022; Tuytschaever et al., 2023). In the present investigation, it was observed that the same trolley units were used for conveying dry-cured filets to the smoking chamber and subsequently for transporting smoked salmon to the pre-packaging area. Along this route, the wheels may come into contact with potential harborage sites located on the floor, thereby facilitating the dissemination of the pathogen to other production areas. Therefore, FBOs should reinforce sanitation procedures, with particular attention to the cleaning and disinfection of trolleys and their structural components.

Moreover, the consistent detection of *L. monocytogenes* on workers’ footwear, drainage channels, and trolley wheels across all three sampling visits, pointing out the need for the smoked salmon processing plant to strengthen control measures targeting frequently contaminated NFCS, with particular attention to these recurrently contaminated sites.

Regarding FCS, *L. monocytogenes* was isolated from trimming tables and in conveyor belts (Tables 2, 3). In this processing plant, the trimming table represents the first surface with which cold-smoked salmon comes into contact upon entering the pre-packaging area (Figure 1). As tables are FCS relatively easy to clean and sanitize (Belias et al., 2022), one possible explanation, which could be investigated through future upstream sampling, is that *L. monocytogenes* may be transferred to this site from early stages of production.

The detection of the pathogen on conveyor belts was not surprising, as these systems are consistently reported among the most common contaminated sites in food processing environments. This is mainly due to the difficulty associated with their effective cleaning and sanitation, which often requires complete disassembly of the equipment (Blatter et al., 2010). Furthermore, conveyor belts commonly present junctions, crevices, and gaskets that constitute ideal niches for microbial proliferation (Belias et al., 2024; Blatter et al., 2010; Carpentier and Cerf, 2011).

The presence of *L. monocytogenes* on trimming table and conveyor belt represents a significant concern for the cold-smoked salmon facility investigated in this study, not only due to the potential contamination of the food product itself but also because of the risk of pathogen dissemination to downstream areas and throughout the entire processing flow.

Concerning the isolation of *L. monocytogenes* from a single food matrix sample (Tables 2, 3), it was observed during the sampling visit carried out in December 2023. As previously mentioned, this period corresponds to a higher production intensity, therefore, potential failures in control strategies may have contributed to contamination of the food matrix. Moreover, this finding may indicate limited transfer to sliced products, possibly due to the presence of salmon competitive microflora, that could restrict pathogen proliferation. Indeed, interactions with background microflora have been shown to limit the growth of *L. monocytogenes*, suggesting that competitive microbial communities can influence its behavior within the food matrix (Lardeux et al., 2015).

The 18 *L. monocytogenes* collected isolates were molecularly characterized and grouped by MLST into five STs: ST5, ST6, ST7, ST9 and ST14. The most prevalent were ST5 (*n* = 7; 38.89%), and ST9 (*n* = 5; 27.78%) followed by ST6 (*n* = 3; 16.67%), ST7 (*n* = 2; 11.11%) and ST14 (*n* = 1; 5.56%). All identified STs profiles have been previously reported and globally distributed (Félix et al., 2022; Maury et al., 2016).

ST9 is one of the most frequently detected genotypes among food-processing isolates, together with ST121 (not detected in this study) (Alvarez-Molina et al., 2021; Maury et al., 2016). Particularly, ST9 is common in facilities processing meat where numerous episodes of persistence have been documented (Fagerlund et al., 2020; Guidi et al., 2022; Melero et al., 2019). Conversely, in fish and seafood sector the presence of ST9 has been observed to a more limited extent (Brauge et al., 2023). Due to its sporadic occurrence among clinical samples, genetic profile ST9 has previously been classified as hypovirulent (Maury et al., 2016).

ST5 has already been associated with industrial environments and with some cases of persistence (Kurpas et al., 2020; Lambrechts et al., 2023; Parra-Flores et al., 2022). Regarding its virulence potential, ST5 has been considered to display an intermediate profile (Maury et al., 2016). So far, it has been implicated in a limited number of listeriosis outbreaks and its frequency among clinical samples remain moderate (Chen et al., 2017; Zhang et al., 2021).

Concerning ST6, it has been described as clinically relevant and has been repeatedly associated with several large-scale outbreaks [Guidi et al., 2023; EFSA (European Food Safety Authority) ECDC (European Centre for Disease Prevention and Control), 2018; Nüesch-Inderbinen et al., 2021]. A notable example is the 2017–2018 South African outbreak, in which ST6 was the predominant type, leading to 567 reported cases of infection (Thomas et al., 2020). The detection of this ST6 within the processing environment may represent a potential food safety concern. However, molecular typing alone does not provide sufficient information to fully assess the pathogenic potential of the isolates. Therefore, future phenotypic characterization combined with whole-genome sequencing (WGS) will be essential to better evaluate the virulence potential of these strains and to investigate their possible association with listeriosis outbreaks.

Finally, ST7 and ST14 have previously been isolated from various food processing facilities, including those handling meat, cheeses and smoked fish (Brauge et al., 2023; Brown et al., 2024; Morganti et al., 2016). Interestingly, ST7 seems to show a geographical association with Northern European countries, where it has frequently been detected in fish processing facilities and it is recognized as one of the most common among clinical cases (Fagerlund et al., 2022; Møretrø et al., 2024; Wagner et al., 2022). In the present investigation, ST7 and ST14 were observed to a limited extent, suggesting a sporadic occurrence of these strains within the cold-smoked salmon processing plant. Future samplings will help monitor the presence of these genotypes over time.

Examining STs distribution, ST5 and ST9 were detected in workers’ footwear during all three sampling visits (Table 3). This observed detection pattern is consistent with the possible persistence of specific *L. monocytogenes* strains or the presence of a recurrent contamination route associated with footwear. Nevertheless, MLST provides limited discriminatory power and does not allow confirmation of clonal relationships among isolates. Future analyses based on WGS and core-genome MLST (cgMLST) will provide information on genetic relatedness and help to clarify whether these recurrent STs represent persistent clones within the processing environment.

Conversely, trolley wheels and drainage channels showed a different temporal distribution pattern, with distinct STs detected across the three sampling visits (Table 3). This variability suggests that contamination at these sites may be driven by repeated introduction events rather than by the long-term persistence of a single strain. These findings highlight the importance of considering both persistent reservoirs and recurrent contamination pathways when designing environmental monitoring and control strategies for *L. monocytogenes* in food processing environments.

## 4 Conclusion

The results of this study highlight the main critical control points within the pre-packaging area of a cold-smoked salmon processing facility, identifying potential hotspots associated with the recurrence and dissemination of *L. monocytogenes*. Environmental monitoring proved to be a valuable tool for assessing the effectiveness of cleaning and sanitation procedures and for detecting potential weaknesses in the hygiene management system. Overall, these findings underscore the need for targeted corrective measures, including enhanced cleaning and disinfection protocols, more frequent disassembly and maintenance of equipment, and strengthened worker hygiene practices.

The use of MLST provided a preliminary overview of the genetic diversity of *L. monocytogenes* strains isolated within the pre-packaging area of the investigated processing plant. Moreover, it enabled the identification of potentially clinically relevant strains and suggested the potential persistence of isolates belonging to two STs. However, these findings should be interpreted in light of the limitations of the present study, including the low number of samples analyzed, the inclusion of a single processing area, and the restricted sampling period. Future studies should include additional production stages and extend monitoring periods to better characterize the distribution and persistence of *L. monocytogenes* within the facility.

In addition, future WGS-based analyses will expand the genetic characterization of *L. monocytogenes* strains by enabling the identification of virulence-associated and stress-resistance determinants. Furthermore, cg-MLST will provide higher-resolution information on strain relatedness, allowing the hypothesis of persistence to be further investigated and confirmed or refuted.

## Data Availability

The original contributions presented in the study are included in the article/supplementary material, further inquiries can be directed to the corresponding author/s.
